# Correction: The effect of job and personal demands and resources on healthcare workers’ wellbeing: A cross-sectional study

**DOI:** 10.1371/journal.pone.0342440

**Published:** 2026-02-10

**Authors:** The PLOS One Editors

## Notice of Republication

This article [1] was republished on July 3, 2024, to address an issue identified post-publication. An updated version of S1 File is provided with this notice. Please download this article again to view the correct version.

The article’s Data Availability statement is updated to: All relevant data are within the paper and the Supporting Information files.

## Supporting information

S1 File Minimal dataset. (XLSX)
