# Supplementary material for: The effect of job and personal demands and resources on healthcare workers’ wellbeing: A cross-sectional study
Source: PLoS One. 2024 May 29;19(5):e0303769. doi: 10.1371/journal.pone.0303769 (PMC11135754; doi:10.1371/journal.pone.0303769)
Supplement: Attachment — Submitted filename: PLOS One_PONE-D-23-27017 Response to reviewers.docx [file pone.0303769.s001.docx]

**Reviewer Comments**

We would like to thank the reviewers for their careful and thorough consideration of this manuscript and for the thoughtful comments and constructive suggestions. We hope our revisions have improved the paper to your satisfaction. Our responses to your comments are below.

**Journal Requirements**

1. Please ensure that your manuscript meets PLOS ONE's style requirements, including those for file naming.

**Response:** The manuscript was revised to meet the style requirements.

2. If you’ve not already done so, consider depositing your raw data in a repository to ensure your work is read, appreciated and cited by the largest possible audience.

**Response:** We are providing the minimal data set with the submission.

3. We noticed you have some minor occurrence of overlapping text with the following previous publications (but not limited to these), which needs to be addressed.

**Response:** This has been addressed.

4. Please confirm at this time whether or not your submission contains all raw data required to replicate the results of your study. Authors must share the “minimal data set” for their submission.

**Response:** We have shared the “minimal data set” this time.

5. Please ensure that you include a title page within your main document.

**Response:** The title page has been added.

6. Please amend your manuscript to include your abstract after the title page.

**Response:** The abstract has been added after the title page.

**Additional Editor Comments**

7. Abstract: Clarify the discrepancy between mentioning a single hospital and describing a multicenter study. Provide more details on the sample size recruitment procedure, such as the use of convenience sampling

**Response:** The multicenter was removed and convenience sampling was added.

8. Explicitly state the unknown aspects covered by your study in each paragraph. Clearly articulate the specific individual and job characteristics you aim to investigate. Include the main aim of the study, as outlined in the JD-R model, in the abstract section.

**Response:** Abstract has been rewritten to address the issue raided in the comment.

9. Methods: Elaborate on how you ensured that healthcare professionals from other areas of KSA responded to your recruitment efforts. Specify the sampling method used. Clarify whether the study includes multiple cities, with a mention of Dammam. Indicate the language used in the survey (Arabic or English).

**Response:** Additional details were added in the “Recruitment” section: “Data were collected through an anonymous online survey in English using Google Forms between February and March 2022. The survey link was shared through email invitations sent through the organization’s listserv for healthcare workers in all cities (Dammam, Al Ahsa, Riyadh, Madinah, and Jeddah) and through WhatsApp messages sent to personal contacts of two of the authors.”

**Under “Study Participants” the following statement was added:** “Convenience sampling was used to recruit participants from all cities.”

10. Results: Provide explanations for symbols in Table 1. Mention abbreviations before their first use. Include significance values (P <0.001**) in Table 3.

**Response:** Table 1 contains only two symbols ‘N’ and ‘n’. ‘N’ denotes sample size, whereas, ‘n’ donates sub-samples in each group. We have added a note to Table 1 to explain these.

The abbreviations used in Table 3 are described in the note after the table. We have added p value to the title of Table 3.

11. Discussion: Begin the discussion with a paragraph highlighting the key results without repeating the aim. Expand on the unique aspects of KSA healthcare professionals contributing to burnout or other studied factors. Ensure clarity and readability of figures

**Response:** In line with the comment, we have restructured the discussion into sub-sections of key findings, theoretical and practical implications, and limitations.

**Reviewer 1**

12. Overall it is good research and contribution to the existing body of literature based on empirical analysis and comprehensive discussion, however the implications of the study are not discussed in explicit way.

**Response:** We have explained theoretical and practical implications.

**Reviewer 2**

13. The current manuscript aims to analyze the relationship of various individual and job characteristics with regards to employee wellbeing in a population of healthcare workers in a government hospital in the Kingdom of Saudi Arabia. The paper should be accepted for publication after some clarifications: - please define the categories of health workers (healthcare professionals, health associate professionals, personal care workers, health management and support personnel, and health service providers not elsewhere classified) and their workplace tasks and activities,

**Response:** The categories were clarified, and a reference was added to the WHO classification of healthcare workers.

14. It is good to list the hypotheses in a similar fashion as they are shown in the other parts of the manuscript (workload, loneliness, organization support, and resilience as antecedents; burnout and work engagement as mediators; and wellbeing as a dependent variable)

**Response:** We have updated the hypotheses to look coherent in the hypotheses section

15. Please describe the importance of the findings for the practical work and implementation of preventive activities

**Response:** We have now included both the theoretical and practical implications of the study.
